# Neurogenic Bladder: Epidemiology, Diagnosis, and Management

**DOI:** 10.1055/s-0040-1713876

**Published:** 2020-10-16

**Authors:** Jalesh N. Panicker

**Affiliations:** 1 Department of Uro-Neurology, The National Hospital for Neurology and Neurosurgery and UCL Queen Square Institute of Neurology, London, United Kingdom

**Keywords:** neurological disease, incontinence, botulinum toxin, tibial nerve stimulation

## Abstract

Lower urinary tract dysfunction is a common sequel of neurological disease resulting in symptoms that significantly impacts quality of life. The site of the neurological lesion and its nature influence the pattern of dysfunction. The risk for developing upper urinary tract damage and renal failure is considerably lower in patients with slowly progressive nontraumatic neurological disorders, compared with those with spinal cord injury or spina bifida. This acknowledged difference in morbidity is considered when developing appropriate management algorithms. The preliminary evaluation consists of history taking, and a bladder diary and may be supplemented by tests such as uroflowmetry, post-void residual measurement, renal ultrasound, (video-)urodynamics, neurophysiology, and urethrocystoscopy, depending on the clinical indications. Incomplete bladder emptying is most often managed by intermittent catheterization, and storage dysfunction is managed by antimuscarinic medications. Intra-detrusor injections of onabotulinumtoxinA have revolutionized the management of neurogenic detrusor overactivity. Neuromodulation offers promise for managing both storage and voiding dysfunction. In select patients, reconstructive urological surgery may become necessary. An individualized, patient-tailored approach is required for the management of lower urinary tract dysfunction in this special population.

The term “neurogenic bladder” is loosely used to denote lower urinary tract (LUT) dysfunction caused by neurological disease. LUT dysfunction is commonly reported by patients with neurological disease, and the impact on quality of life is being increasingly recognized by the practicing neurologist. Its heterogeneous presentation reflects the complexity of the neural control of the LUT, and the site of lesion in the neurological axis determines the pattern of symptoms and dysfunction. Pelvic organ dysfunction encompasses LUT, sexual, and bowel dysfunction, and their complex interrelationship is now better understood, with the recognition that a holistic approach is required for management. This review presents an overview of neurological control of LUT functions in health, a clinical approach to evaluating LUT dysfunction in the context of neurological disease, and an overview of current treatment strategies.

## Neurological Control of Lower Urinary Tract Functions in Health

A complex neural network acts as a switching circuit to maintain a reciprocal relationship between the reservoir function of the bladder and sphincter function of the urethra. This results in low-pressure filling and periodic voluntary emptying. The frequency of micturition in a person with a bladder capacity of 400 to 600 mL is once every 3 to 4 hours, and the bladder is in a storage phase more than 99% of the time. 1 2 Switching from the storage to the voiding phase is initiated by a conscious decision, which is influenced by the perceived state of bladder fullness and an assessment of the social appropriateness of voiding. This phasic pattern of activity, as well as degree of voluntary control and dependence on learned behavior, is unique to the LUT compared with other autonomically innervated structures such as the cardiovascular system. 1

Connections between the pons and the sacral spinal cord, as well as the peripheral innervation that originates from the caudal spinal cord, need to be intact to effect storage and voiding. During bladder filling, sympathetic and pudendal nerves mediate contraction of the smooth (internal) and striated (external) urethral sphincter, whereas sympathetic-mediated inhibition of the detrusor prevents contractions. This results in low pressure filling and continence. 1 When deemed appropriate to void, the pontine micturition center (PMC) is released from the tonic inhibition of higher cortical centers, and parasympathetic-mediated detrusor contraction accompanied by relaxation of the pelvic floor and external and internal urethral sphincters results in effective bladder emptying. 3 4

## Neurogenic Lower Urinary Tract Dysfunction

**Fig. 1:**
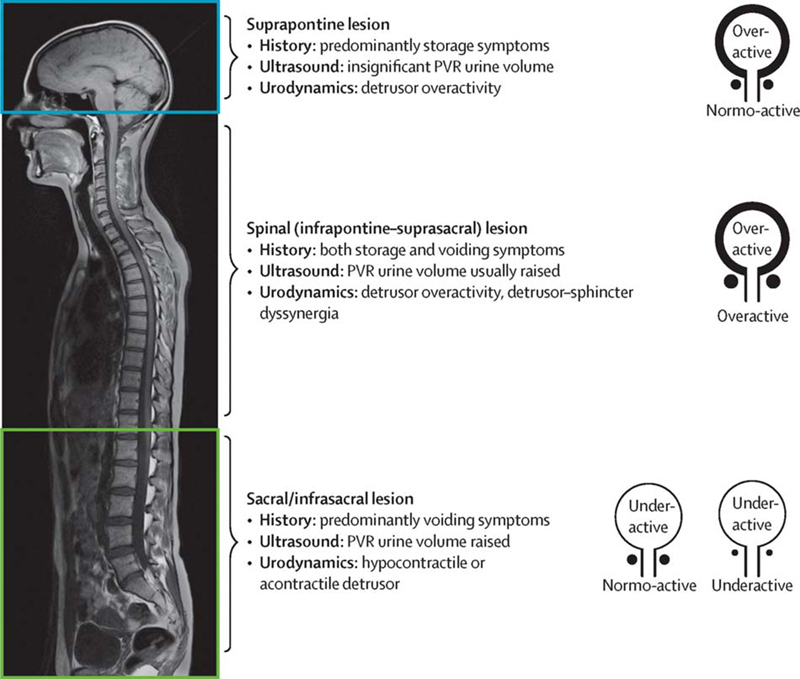
The pattern of lower urinary tract dysfunction following neurological disease is influenced by the site of lesion. PVR, post-void residual. (Used with permission from Panicker et al. 5 )

**Fig. 2:**
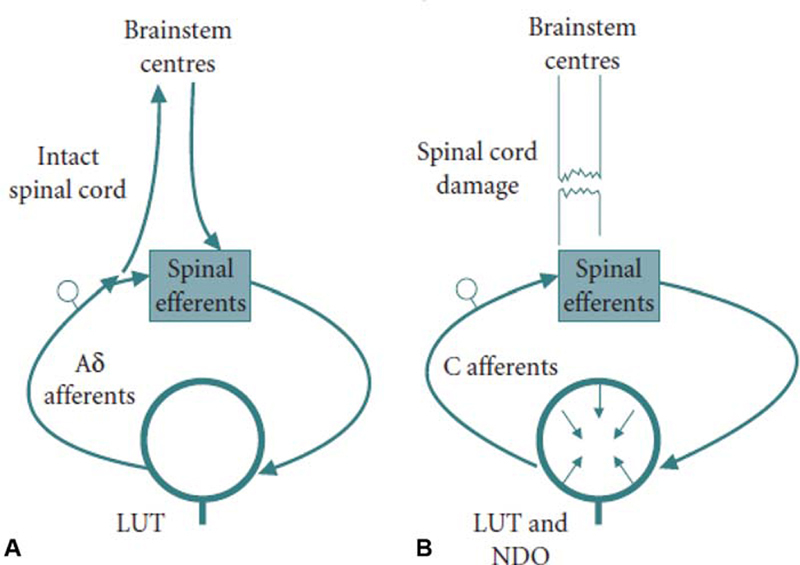
Two types of afferent nerves convey sensations of bladder filling. ( **A** ) In health, thinly myelinated Ad fibers have a lower threshold for activation and are responsible for conveying sensations of bladder filling, whereas unmyelinated C-fibers have a greater threshold for activation and are thought to be quiescent. ( **B** ) Following spinal cord damage, C-fibers become sensitized and are mechanosensitive at lower bladder volumes. A segmental spinal reflex emerges that is mediated by C-fibers afferents nerves and results in involuntary detrusor contractions, the basis for NDO. LUT, lower urinary tract; NDO, neurogenic detrusor overactivity. (Used with permission from Panicker et al. 7 )

A neurourological classification of neurological disorders is useful to understand that different patterns of LUT dysfunction can arise following neurological disease ( Fig. 1 ). 5 *Detrusor overactivity* (DO) is the most common cause for urinary incontinence following neurological disease. Patients report varying degrees of storage symptoms such as urinary urgency, frequency, nocturia, and incontinence (collectively known as “overactive bladder symptoms”). Damage to central inhibitory pathways or sensitization of peripheral afferent terminals in the bladder can unmask primitive voiding reflexes and be expressed as spontaneous involuntary contractions of the detrusor. 6 The mechanisms for detrusor overactivity following suprapontine damage are, however, different from those following spinal cord injury (SCI). A lesion affecting the suprapontine neural network results in removal of the tonic inhibition of the PMC and involuntary detrusor contractions. Detrusor overactivity occurring following a lesion of the suprasacral spinal cord is mediated through the emergence of a spinal reflex pathway that triggers bladder overactivity. Afferent nerves conveying sensations from the LUT to the spinal cord contain unmyelinated C-fibers that have a much greater threshold for activation and are therefore quiescent in health. Following SCI, the C-fibers become sensitized and are mechanosensitive at lower bladder volumes. 1 It has been shown in experimental animal models of SCI that a C-fiber afferent-mediated segmental spinal reflex emerges, resulting in involuntary detrusor contractions at low bladder volumes ( Fig. 2 ). 7 8 Additionally, normally coordinated activity between the detrusor and urethral sphincters during voiding becomes impaired, and the detrusor and urethral sphincters contract simultaneously, termed *detrusor-sphincter dyssynergia* (DSD). Injury to the conus medullaris, sacral roots (cauda equina), and peripheral nerves results primarily in voiding dysfunction from poorly sustained detrusor contractions, termed *detrusor underactivity* . Patients with DSD or detrusor underactivity report different voiding symptoms that include urinary hesitancy and an interrupted urinary stream, sensation of incomplete bladder emptying and double voiding. Variations from the expected patterns of LUT dysfunction, presented in Fig. 1 , should warrant a search for additional urological pathologies, or even neurological sites of localization.

### Parkinson's Disease and Multiple System Atrophy

The prevalence of LUT symptoms in Parkinson's disease ranges between 38 and 71%, depending on the extent and duration of disease. LUT symptoms are the commonest nonmotor symptoms reported in PD, 9 and are associated with an increased risk for falls, 10 early institutionalization, and escalating health-related costs. 11 Nocturia is the most common LUT symptom and can arise due to impaired bladder storage from DO and from nocturnal polyuria. 12 13

In multiple system atrophy (MSA), LUT symptoms occur early in the course, often preceding other neurological manifestations and even orthostatic hypotension. 14 Incontinence arises from detrusor overactivity and external sphincter weakness; however, as the disease progresses, incomplete bladder emptying becomes the predominant feature. 15 The finding of an open bladder neck in video-urodynamic studies in men who have not undergone surgery to the bladder outlet is a suggestive sign of sympathetic denervation of the bladder neck seen in MSA. 16

### Dementia

Incontinence is often a prominent symptom and contributes significantly to caregiver's burden. Incontinence occurs early in normal pressure hydrocephalus, dementia with Lewy's bodies, vascular dementia, and frontotemporal dementia, whereas it generally occurs late in the course of Alzheimer's disease or Parkinson's disease with dementia. 17 18 In patients with dementia and incontinence, pharmacological treatment of one condition can exacerbate the other. 19 Incontinence may result not only from detrusor overactivity, but also from cognitive and behavioral problems, urological comorbidities, and immobility.

### Cerebrovascular Disease

More than half of stroke patients report urinary incontinence during the acute phase of stroke. Risk factors for incontinence include large lesion size and the presence of comorbid illnesses such as diabetes and older age. Lesions in the anteromedial frontal lobe, periventricular white matter, and putamen are most commonly associated with LUT symptoms. Most commonly, urodynamic studies show evidence for detrusor overactivity. Urinary retention has been reported following hemorrhagic and ischemic stroke. 20 There is evidence to suggest that incontinence is independently associated with subsequent severity of neurological disability, institutionalization, and mortality. Small vessel disease of the white matter, leukoaraiosis, is associated with urgency incontinence, and it is now apparent that this is an important cause for incontinence in the functionally independent elderly. 21 22 23

### Multiple Sclerosis

Lower urinary tract symptoms are reported in 32 to 96% of patients with MS, depending on the duration and severity of disease. Symptoms are reported on average six years into the illness, and more than 90% of patients report symptoms if the duration of disease exceeds 10 years. 24 Symptoms may be overlooked, however, and the results of a recent survey suggest that nearly half of MS patients with moderate-to-severe overactive bladder symptoms had not undergone an evaluation by a urologist. 25 Relevant to a neurology practice is that 10% of patients report LUT symptoms at the time of initial diagnosis. 24 Most patients classify the impact symptoms have on their life as moderate or high, and urinary incontinence is considered to be one of the worst aspects of the disease. 26 Most commonly, both storage and voiding symptoms are reported. The most common findings in urodynamic testing are DO and DSD. 27

### Spinal Cord Injury

During the stage of spinal shock, patients are often in urinary retention. They develop the typical pattern of DO and DSD over time as spinal reflexes return. Pressures within the bladder may rise considerably, increasing the risk for upper urinary tract damage. Urinary retention, bladder spasms, UTIs, interventions of the bladder and bowel, constipation, and sexual activity can trigger autonomic dysreflexia in individuals with lesions at the T6 spinal cord level or above. 28

### Spina Bifida

Lower urinary tract dysfunction is common in spina bifida, reported in more than 90% of children. Dysfunction begins in utero, and symptoms usually manifest soon after birth, though may be delayed until childhood, and occasionally until adulthood. Urodynamic studies demonstrate a variety of findings including detrusor overactivity, detrusor underactivity or low compliance, DSD, or a static or fixed external urethral sphincter. Young individuals may develop LUT dysfunction later on in life due to spinal cord tethering. They should therefore be regularly monitored, as they may be at risk for developing upper urinary tract complications.

### Cauda Equina Syndrome and Peripheral Neuropathy

Reduced or absent detrusor contractions are commonly seen. Individuals report reduced sensation of bladder fullness, inability to initiate micturition voluntarily, and bladder distension, to the point of overflow incontinence. Detrusor overactivity may, however, occur in a subset of patients, and this may be as a result of “bladder decentralization” due to preserved peripherally sited postganglionic neurons. 29

### Urinary Retention

Urinary retention may occur following neurological disease ( Table 1 ). 30 Medications such as opiates, 31 those with anticholinergic properties (e.g., neuroleptics, tricyclic antidepressants, respiratory agents with anticholinergic effects, and antimuscarinic agents for the bladder) and α-adrenoceptor agonists may cause voiding dysfunction, ranging from incomplete bladder emptying to complete urinary retention.

**Table 1:** The differential diagnosis for urinary retention in a patient where a structural urological lesion has been excluded

1. Lesions of the conus medullaris or cauda equina
Compressive lesions
Spine fracture
Intervertebral disc prolapse
Space-occupying lesions—tumor, granuloma, abscess
Noncompressive lesions
Vascular—infarction, AV malformation with vascular steal
Inflammation—myelitis, meningitis retention syndrome
Infection—herpes simplex, varicella zoster, cytomegalovirus, Elsberg's syndrome (viral aseptic meningitis)
2. Other neurological conditions
Spina bifida
Multiple system atrophy
Autonomic failure, e.g., pure autonomic failure, autonomic neuropathies
3. Miscellaneous
Medications, e.g., opioids, anticholinergics, retigabine
Radical pelvic surgery
Fowler's syndrome (females)

Abbreviation: AV, arteriovenous malformation.

Urinary retention is relatively uncommon in young women, and if no underlying urological or neurological disease can be established, a primary disorder of urethral sphincter relaxation (called Fowler's syndrome) should be considered. Women typically present with painless urinary retention, often with volumes exceeding a liter, in the absence of a sensation of bladder fullness. They often experience difficulty performing intermittent catheterization, especially when attempting to remove the catheter. Urethral sphincter electromyography (EMG) reveals a characteristic pattern of abnormal activity, and the urethral pressure profile is usually elevated. Sacral neuromodulation, and more recently sphincter injections of Botulinum toxin, has been shown to be effective treatments. 32 33

## Impact of Lower Urinary Tract Dysfunction on the Upper Urinary Tract

In some individuals, high pressures within the LUT may affect the upper urinary tract, resulting in vesicoureteric reflux, hydronephrosis, and in some instances renal impairment and end-stage renal disease. Patients with SCI or spina bifida are at significant risk of developing these problems, having a five or eight times risk, respectively, of developing renal failure compared with the general adult population. 34 For reasons that are not fully understood, the prevalence of upper urinary tract damage and renal failure is much lower in patients with slowly progressive nontraumatic neurological disorders. In patients with multiple sclerosis, the risk for urinary tract complications is higher with increasing disease duration and severity of disability, 35 as is also the case for patients with Parkinson's disease and hereditary spastic paraplegia. 36 The management of neurogenic LUT dysfunction should include a risk assessment of developing upper urinary tract damage: in patients at high risk of upper urinary tract damage, management is focused on life-long frequent urological evaluations to define the need for interventions to reduce the risk of complications. Conversely, in patients with progressive nontraumatic neurological disorders, such as Parkinson's disease or multiple sclerosis, who are typically at low risk for upper urinary tract involvement, the management is focused on symptomatic control of neurogenic LUT dysfunction.

## Evaluation

A multidisciplinary approach involving neurologists, urologists, physiatrists, and primary care physicians is key to successful management of LUT dysfunction ( Table 2 ). 5

**Table 2:** Assessment of the patient with neurological disorders reporting LUT symptoms

	Bedside evaluation	Noninvasive tests	Invasive tests
Essential	• History taking • Physical examination • Bladder diary	• Urinalysis • PVR measurement Ultrasonography	N/A
Desirable	Questionnaires	• Uroflowmetry • Blood biochemistry	N/A
Required in specific situations	N/A	• Urine culture • Urine cytology	• (Video-) urodynamics • Cystoscopy • Pelvic neurophysiology • Renal scintigraphy

Abbreviation: LUT, lower urinary tract. (Used with permission from Panicker et al. 5 )

### History Taking and Physical Examination

Information is gathered on LUT symptoms, congenital and neurological abnormalities, prior urogenital complications and treatments, sexual and bowel complaints, and impact on quality of life. 28 Medication history should be reviewed. For example, an association between opiate use and voiding dysfunction is often overlooked despite being a listed adverse effect. 31 Not infrequently, individuals may report becoming incontinent due to the inability to reach the toilet in a timely manner due to their neurological deficits or due to poor accessibility to toilets (functional incontinence).

The bladder diary provides a real-time objective patient-reported measure of LUT symptoms, which may not be obtained through history taking or questionnaires. 37 The diary is ideally maintained for 3 days; however, to be of value, the individual must be motivated to complete it faithfully.

### Investigations

Urinalysis and urine culture (if appropriate) and blood chemistry, if not already performed by the referring physician, form part of a basic assessment.

### Ultrasonography

The post-void residual (PVR) volume is measured by ultrasound or in–out catheterization. An elevated PVR signifies that there is voiding dysfunction; however, it does not discern whether this is due to detrusor underactivity or bladder outflow obstruction, for which urodynamic studies would be required. The PVR should ideally be measured on different occasions, as the degree of bladder emptying varies at different times and in different circumstances. 38 39

In patients known to be at high risk of upper urinary tract disease, ultrasonography should be performed periodically to screen for upper urinary tract dilatation or renal scarring. 28 Ultrasound may also be used to demonstrate urinary tract stones, which may develop in patients with neurogenic LUT dysfunction.

### Urodynamic Investigations

#### Noninvasive Urodynamic Tests

Uroflowmetry provides a valuable noninvasive assessment of voiding functions, and the PVR is usually measured with the test. The pattern and rate of flow depend on detrusor function and bladder outlet resistance.

#### Invasive Urodynamic Tests

Combined cystometry and pressure-flow study, with or without simultaneous fluoroscopic monitoring (i.e., video-urodynamics), assess detrusor and bladder outlet function and provide information about detrusor pressure and compliance. Urodynamic testing helps establish the pathophysiological basis for LUT symptoms, but also serves as a tool to assess risk for upper tract damage as discussed earlier.

The place of urodynamics in the evaluation of LUT symptoms in neurological patients is a matter of ongoing debate. 38 For example, in the assessment of LUT functions in patients with early multiple sclerosis, urodynamic studies are recommended by the French guidelines, 24 whereas in the United Kingdom, the recommended first-line management involves testing for urinary tract infections (UTIs) and PVR measurement without invasive urodynamics. 39 In the absence of studies comparing these two models, the decision to perform complete baseline urodynamic studies would depend on local recommendations and resources.

### Other Tests

Other LUT pathologies may coexist with LUT symptoms, and cystoscopy (combined with bladder washing cytology if appropriate) is indicated when urethral stricture, bladder stones, and bladder tumors are suspected. 28

Measuring serum creatinine and calculating the estimated glomerular filtration rate (GFR) yield a reasonable estimation of renal function with minimal cost and inconvenience. The GFR is most accurately measured using renal scintigraphy, which is recommended when renal function is poor, in individuals with reduced muscle mass, if function for each renal unit has to be assessed separately and in high-risk patients. 28

### Pelvic Neurophysiology

The role of pelvic EMG in the assessment of individuals with established neurological disease is limited. Pelvic floor EMG was first introduced as part of urodynamic studies with the aim of recognizing DSD. However, it is less commonly performed nowadays with the advent of video-urodynamics testing. Sphincter muscle EMG is useful in evaluating the innervation of the sacral second, third, and 4th innervation, when cauda equina syndrome is suspected, 40 or sometimes in patients presenting with a parkinsonian syndrome to aid in the differential diagnosis between idiopathic Parkinson's disease and MSA. 41 Urethral sphincter EMG has proven to be useful in the evaluation of young women in urinary retention when Fowler's syndrome is suspected. 42

## Management

The goals of management are to achieve urinary continence, improve quality of life, prevent UTIs, and preserve upper urinary tract function. 28 43 The management of neurogenic LUT dysfunction should address both voiding and storage dysfunction, and is influenced by the severity of symptoms and risk for developing upper urinary tract damage. Adopting an approach that includes evaluating LUT symptoms, risk assessment for upper tract impairment, and regular review after instituting treatment provides a framework for the practicing neurologist to manage LUT dysfunction in their patients with progressive nontraumatic neurological disorders. Table 3 presents the different treatment options. 5 There are situations where specialist urology services should be involved early in the care of these patients ( Table 4 ).

**Table 3:** Treatment options for bladder dysfunction

	Storage dysfunction		Voiding dysfunction
	Urgency, frequency ± incontinence	Stress incontinence	
Conservative	• Behavioral therapy • Antimuscarinic agents • Desmopressin • OnabotulinumtoxinA into the detrusor • Beta-3-receptor agonists • Tibial neuromodulation	Pelvic floor muscle exercises	• Intermittent catheterization • Indwelling catheterization • Triggered voiding • Alpha-blockers • OnabotulinumtoxinA into the external sphincter
Surgical	• Sacral neuromodulation • Bladder augmentation • Sacral deafferentation/anterior root stimulation	• Bulking agents • Autologous/synthetic slings • Balloons • Artificial sphincter	• Sacral neuromodulation • Intraurethral stents • External sphincter/bladder neck incision • Transurethral resection of prostate
	Continent/incontinent urinary diversion		

(Used with permission from Panicker et al. 5 )

**Table 4:** Red flags warranting a referral to a urology service

• Hematuria
• Recurrent urinary tract infections
• Pain suspected to be originating from the urinary tract
• Increased risk for upper urinary tract damage, or findings of upper tract damage or renal impairment in imaging or blood tests
• Suspicion of concomitant urological or gynecological pathologies, e.g., bladder outlet obstruction due to prostate enlargement in men or stress incontinence in women
• Lower urinary tract symptoms refractory to conservative treatments—consideration of more invasive treatments such as intradetrusor injections of botulinum toxin A or surgery, or when suprapubic catheterization appears appropriate

## Managing Storage Dysfunction

### Antimuscarinic Agents

Competitive antagonism of the muscarinic receptors results in detrusor relaxation and lower intravesical pressures. Since the introduction of oxybutynin, several newer antimuscarinic agents have appeared on the market ( Table 5 ) and have been shown to be efficacious in neurogenic OAB. 44 45 46 47 48 The findings of systematic reviews suggest that the only difference between medications is their side effect profile. 49 50 Prescribing patterns of these agents are determined by local guidance.

**Table 5:** Commonly used antimuscarinic agents

Antimuscarinic agent	Preparation	Dosage (mg)	Frequency
Darifenacin	Controlled release	7.5–15	Once daily
Fesoterodine	Controlled release	4–8	Once daily
Oxybutynin	Immediate release	2.5–5	Two or three times a day
	Controlled release	5–20	Once daily
	Transdermal patch	36, releasing oxybutynin ~3.9 mg/24 h	Replace once every 3–4 d
Propiverine	Immediate release	15	One to three times daily
	Controlled release	30	Once daily
Solifenacin	Controlled release	5–10	Once daily
Tolterodine	Immediate release	2–4	Once or twice daily
	Controlled release	4	Once daily
Trospium chloride	Immediate release	20	Twice daily (before food)
	Controlled release	60	Once daily

The common adverse effects include dry mouth, blurred vision for near objects, constipation, and occasionally tachycardia. These symptoms are often reported by individuals already with neurological disease and therefore must be reviewed before commencing the medication. The PVR may increase after starting an antimuscarinic agent, and therefore should be monitored in case of a poor response to treatment or paradoxical worsening of symptoms. 39

Several of the antimuscarinic agents can cross the blood–brain barrier, and through their effects on central muscarinic receptors can result in adverse effects such as alterations in cognition and consciousness. Increasing the anticholinergic burden from cumulative use of medications with anticholinergic properties may be associated with worsening cognitive functions, MRI findings of brain atrophy, decline in physical functions, and falls. 51 52 53 In susceptible individuals such as the elderly, it would appear sensible to recommend the use of an antimuscarinic agent with minimal effects on the central muscarinic receptors. Trospium chloride is relatively impermeable to the blood–brain barrier because of its physicochemical properties, and darifenacin has greater affinity for the M3 receptor subtype of relevance to the LUT compared with the M1 subtype, which is prevalent in the brain. 54 55 56 However, evidence supporting their use in clinical practice based on this consideration is limited. 57

### Beta-3-Adrenergic Receptor Agonists

The β-3-adrenergic receptor agonist mirabegron has been approved for managing overactive bladder symptoms. Although devoid of the adverse effects reported with antimuscarinic agents, side effects do occur, including palpitations, hypertension, and, rarely, atrial fibrillation. 58 Studies suggest that mirabegron is efficacious in patients with Parkinson's disease, MS, and SCI, with tolerable side effect profiles. 59 60 61

### Desmopressin

Desmopressin, a synthetic analogue of arginine vasopressin, promotes fluid re-absorption at the distal and collecting tubules of the kidney and thereby temporarily reduces urine production and volume-related detrusor overactivity. It is useful for the treatment of urinary frequency or nocturia in patients with MS, providing symptom relief for up to 6 hours. 62 It is also helpful in managing nocturnal polyuria, a disorder seen in patients with Parkinson's disease and also various neurological conditions associated with orthostatic hypotension. However, it should be prescribed with caution due to the high risk of severe hyponatremia and congestive heart failure.

### Botulinum Toxin

Since the first reports of the use of botulinum toxin injections into the detrusor in patients with SCI, 63 64 several studies have been performed demonstrating the treatment to be highly effective, safe, and well tolerated, culminating in two pivotal phase 3 studies of onabotulinumtoxinA. 65 66 Benefits seem to occur regardless of the underlying neurological disorder; however, data on conditions other than SCI and MS are scarce. A dose of 200 units of onabotulinumtoxinA is injected in the bladder wall, requiring cystoscopy, an intervention that can be performed under local anesthesia in most neurological patients. Long-term data confirm the efficacy of repeat onabotulinumtoxinA injections, 67 68 and cost-effectiveness is superior to best supportive care. 38

### Neuromodulation

Electrical stimulation of the sacral nerve roots, tibial nerve, pudendal nerve, and dorsal genital nerves has been shown to be effective in managing the idiopathic overactive bladder. Electrical stimulation of the tibial nerve has been shown to be a safe and effective treatment for LUT storage symptoms and for bowel dysfunction. 69 Stimulating the nerve using a fine gauge needle (percutaneous tibial nerve stimulation, PTNS) has been shown to improve overactive bladder symptoms and urodynamic parameters in patients with MS and PD. 70 71 A typical treatment course consists of using a fixed frequency electrical signal for 30 minutes over the course of 12 sessions. PTNS is a minimally invasive option for managing patients with mild/moderate overactive bladder symptoms, and is associated with few adverse effects. Moreover, it is one of the few options for the overactive bladder not associated with worsening voiding dysfunction and incomplete bladder emptying. 72 However, effects are relatively short lived, and the need to return for maintenance top-up treatments is influenced by the degree of improvement of LUT symptoms. 73 Alternatively, the nerve can be stimulated using a cutaneous electrode (transcutaneous tibial nerve stimulation), and therefore may be performed at home. This treatment has been shown to be safe and effective in patients with MS or stroke experiencing urgency incontinence. 74 75

## Managing Voiding Dysfunction

Voiding dysfunction may not be apparent from history, and measuring the PVR therefore is integral. A significantly high residual urine after voiding may exacerbate detrusor overactivity, thereby worsening storage symptoms and rendering treatments such as antimuscarinics and botulinum toxin less effective. Moreover, a high PVR volume predisposes the patient to recurrent UTIs. 76 If the PVR is found to be consistently elevated, catheterization, preferably intermittently, is indicated and greatly improves management. 43 The PVR volume at which intermittent self-catheterization should be initiated lacks consensus, however. Many patients with neurogenic LUT dysfunction have a reduced bladder capacity, and therefore a PVR consistently more than 100 mL is often taken as the amount of residual urine that warrants treatment if the patient is symptomatic. 39 A trained health care professional such as a continence advisor should assess whether neurological deficits (poor manual dexterity, weakness, tremor, rigidity, spasticity, impaired visual acuity, or cognitive impairment) may be barriers to successful catheterization. 77 The frequency of catheterization depends on several factors, such as bladder capacity, fluid intake, PVR, and urodynamic parameters. Patients in complete urinary retention will need to catheterize four to six times per 24 hours. The incidence of symptomatic UTIs is low when performed regularly. 39 76 In patients not suitable for intermittent catheterization, a urethral or (preferably) suprapubic indwelling catheter would need to be considered.

Triggered reflex voiding can occasionally be achieved by provoking a bladder contraction, such as suprapubic tapping and thigh scratching, and is most successful in patients with a suprasacral spinal cord lesion. However, these maneuvers should be used with caution as they may provoke a rise in intravesical pressures. 28 Likewise, bladder expression using Valsalva or Credé maneuvers is usually not recommended. 28 Suprapubic vibration using a mechanical “buzzer” 78 and the use of α-adrenergic blockers have been advocated by some groups. 39 Botulinum toxin injections into the external urethral sphincter may improve bladder emptying in patients with SCI who have significant voiding dysfunction. 79

### Surgical Treatment

Surgical interventions should be considered in cases where these first- and second-line treatments have failed. Nowadays, surgery is increasingly becoming uncommon for progressive nontraumatic neurological conditions due to the availability of less invasive options ( Table 3 ). 5

## Follow-up of Patients with Neurogenic Lower Urinary Tract Dysfunction

In the absence of long-term natural history studies, there is a lack of consensus about how often patients with neurogenic LUT dysfunction should be followed up. The population with high risk for upper tract damage should be followed up more regularly, with a patient-tailored approach aiming to achieve an optimal quality of life and to protect the upper urinary tract. Follow-up should reflect regional guidelines based on resource allocation. 28

## Urinary Tract Infections in Neurological Patients

Recurrent UTIs, defined as more than two UTIs in 6 months, or more than three in a year, commonly occur in neurological patients and are an important cause for hospital admissions. It is important to distinguish recurrent infections from persisting infections, as undertreatment of the latter may result in chronic infections. A high PVR can predispose to UTIs, and the incidence of symptomatic UTIs often falls when intermittent catheterization is performed regularly.

An evaluation would include assessing for incomplete bladder emptying (e.g., bladder outflow obstruction, reduced detrusor contractility) or a structural abnormality (foreign body in bladder, bladder stone, tumor, etc.), and for this reason the input of a urologist would be valuable. In individuals with proven recurrent UTIs and in whom no urological structural abnormality has been identified, it would be reasonable to initiate nonantibiotic options such as cranberry extract tablets or D-mannose. 80 Prophylactic low-dose antibiotics for a finite duration may be required; rotating between antibiotics is one approach for minimizing the development of antibiotic resistance. 76 The benefit of cranberry juice in preventing UTIs in neurogenic patients is debatable. 81

## Conclusion

LUT dysfunction is common in neurological patients and has a significant impact on quality of life. Neurologists are increasingly enquiring about LUT functions and becoming involved in the management of these complaints. However, collaboration with other specialists including urologists is highly recommended to maximize patients' quality of life.
